# Effect of freeze–thaw manipulation on phytostabilization of industrially contaminated soil with halloysite nanotubes

**DOI:** 10.1038/s41598-023-49698-7

**Published:** 2023-12-13

**Authors:** Maja Radziemska, Mariusz Z. Gusiatin, Agnieszka Cydzik-Kwiatkowska, Aurelia Blazejczyk, Grzegorz Majewski, Iwona Jaskulska, Martin Brtnicky

**Affiliations:** 1 Institute of Environmental Engineering, Warsaw University of Life Sciences, 02-776 Warsaw, Poland; 2 Faculty of Geoengineering, University of Warmia and Mazury in Olsztyn, 10-719 Olsztyn, Poland; 3 Institute of Civil Engineering, Warsaw University of Life Sciences, 02-776 Warsaw, Poland; 4 Faculty of Agriculture and Biotechnology, Bydgoszcz University of Science and Technology, 85-796 Bydgoszcz, Poland; 5 Department of Agrochemistry, Soil Science, Microbiology and Plant Nutrition, Mendel University in Brno, 613 00 Brno, Czech Republic

**Keywords:** Environmental sciences, Solid Earth sciences

## Abstract

The latest trends in improving the performance properties of soils contaminated with potentially toxic elements (PTEs) relate to the possibility of using raw additives, including halloysite nanotubes (HNTs) due to eco-friendliness, and inexpensiveness. *Lolium perenne L*. was cultivated for 52 days in a greenhouse and then moved to a freezing–thawing chamber for 64 days. HNT addition into PTE-contaminated soil cultivated with grass under freezing–thawing conditions (FTC) was tested to demonstrate PTE immobilization during phytostabilization. The relative yields increased by 47% in HNT-enriched soil in a greenhouse, while under FTC decreased by 17% compared to the adequate greenhouse series. The higher PTE accumulation in roots in HNT presence was evident both in greenhouse and chamber conditions. (Cr/Cd and Cu)-relative contents were reduced in soil HNT-enriched-not-FTC-exposed, while (Cr and Cu) in HNT-enriched-FTC-exposed. PTE-immobilization was discernible by (Cd/Cr/Pb and Zn)-redistribution into the reducible fraction and (Cu/Ni and Zn) into the residual fraction in soil HNT-enriched-not-FTC-exposed. FTC and HNT facilitated transformation to the residual fraction mainly for Pb. Based on PTE-distribution patterns and redistribution indexes, HNT’s role in increasing PTE stability in soils not-FTC-exposed is more pronounced than in FTC-exposed compared to the adequate series. *Sphingomonas*, *Acidobacterium*, and *Mycobacterium* appeared in all soils. HNTs mitigated FTC’s negative effect on microbial diversity and increased *Planctomycetia* abundance.

## Introduction

The constantly increasing amount of soil contaminated with various potentially toxic elements (PTEs) is strictly connected, among others, with the development of industry, progressing urbanization as well as activities connected with agriculture and mining^1^. In the ecological sense, increased concentration of PTEs in the surface layer, including the arable layer, of soils, is a threat, since there is a risk of PTEs being absorbed by the root system and accessing the above-ground parts of plants^2^. The reactions of plants that are grown or occur naturally in contaminated areas also reveal characteristic signs of the toxic effects of these PTEs. Moreover, the danger connected with the presence of PTEs in the soil is connected with the possibility of them spreading to surface and ground waters and their contamination^3^.

The process of returning usable or environmental characteristics to degraded areas can be achieved, among others, by applying the technique of aided phytostabilization, which relies on applying adequately selected plants and soil additives in areas where high PTE contents have been noted^4^. In this case, the grass species *Lolium perenne* L., which, due to its many-year growth cycle and branched-out root system stabilizes the structure of the soil and is fairly resistant to high concentrations of PTEs predominantly as potentially harmful cations, may prove useful^5^.

The application of halloysite nanotubes (HNTs) as an additive aiding the immobilization process of PTEs in soil, which is a raw material falling into the subcategory of kaolinite, which belongs to the group of kaolinite-serpentine, may turn out to be an innovative low-price and optimal-performance solution^6^. It is characterized by a nanotubular construction, large specific surface area, high porosity, ion exchange capacity plus cation exchange selectivity, surface hydrophilicity, and surface electronegativity^7,8^. An additional widely recognized advantage is the low impact of moisture on its durability, which in practice means maintaining efficiency for a long time.

The frozen ground comprises approx. 79% of the total land surface, including 14% permafrost and 56% seasonally frozen soil^9^. The course of aided phytostabilization under changeable temperature conditions has not been sufficiently analyzed to date. There are only a few articles written by Radziemska et al.^2,10^ connected with the influence of freezing and thawing cycles (FTC) on the efficiency of aided phytostabilization. Seasonally frozen soil is subjected to great fluctuations, both temporal as well as spatial. The influence of below-zero temperatures on the soil is varied, influencing a wide range of physical, chemical, and biological processes^11^. The part of the soil which is generally subjected to seasonal freezing and thawing, reaches anywhere from a few centimeters to approx. a meter or even a few meters below the surface. This covers mainly the unsaturated zone, in which the humidity and temperature of the soil react to the dynamics of the atmosphere in a relatively short period, usually from a few hours to a few days^12^.

Keeping the above in mind, studies aimed to determine the influence of the addition of halloysite nanotubes (HNTs) commercially exploited in Poland and variable temperature conditions (FTC) on the efficiency of immobilizing Cd, Cr, Cu, Ni, Pb and Zn during aided phytostabilization. The crop yield of *Lolium perenne* L. was determined, as well as the chemical composition of the biomass of above-ground parts and roots; selected physical and chemical properties of the soil and the structure of the soil microbiome were also analyzed.

## Results and discussion

### HNT and FTC effects on the plant biomass and PTE content

**Figure 1:**
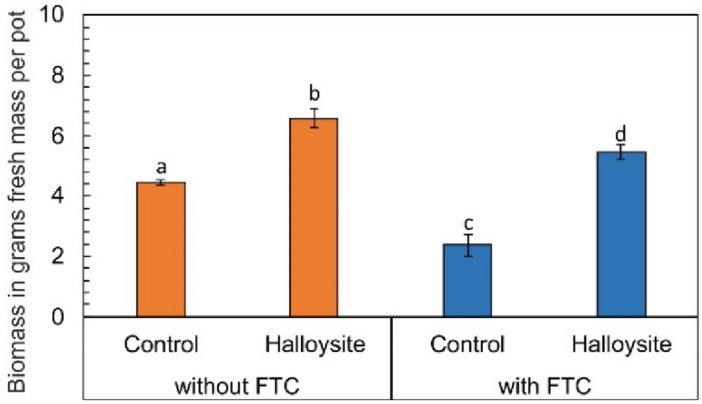
Changes in above-ground biomass in not enriched (control) and halloysite-enriched pots, without and with FTC. Data are shown as mean ± SD, n = 3. Different letters indicate significant differences (*p* < 0.05) in biomass yield from different treatments.

The degree of immobilization and decreasing the bioavailability of PTEs in soils from contaminated areas using the technique of aided phytostabilization depends, among others, on the quality of vegetation cover^13^ as well as the type of introduced soil additive^14^. In the carried out experiment of aided phytostabilization, *Lolium perenne* L. was used, having turned out to be a plant not very sensitive to high concentrations of PTEs in the soil, which is confirmed by, among others, the amount of above-ground biomass. The collected crop yield turned out to be (*p* < 0.05) dependent on the addition of HNTs to the soil as well as on FTC (Fig. 1). In the case of series derived from a greenhouse, in soil enriched with HNTs, plant yield increased by 47% in relation to the control series. The same trend has been reported in previous studies in the application of halloysite to metal-contaminated substrate derived from the military area. The authors observed nearly a twofold increase in the crop yield of *Festuca rubra* L.^15^. Concerning series derived from the freezing–thawing chamber, in soil enriched with HNTs, a similar dependency was observed as in the case of greenhouse series, though the amount of biomass obtained in absolute values was respectively lower. Regarding the effect of FTC exposure on plant yield a more intense relative decrease in plant above-ground parts was observed in the control series by 46% (in relation to the series not enriched with HNTs and not exposed to FTC) than in the HNT series by 17% (in relation to the series enriched with HNTs and not exposed to FTC), showing that low temperatures unfavorably affect crop production and quality but the additive presence in soil can maintain and support the yield even during freezing and thawing.

Under the influence of exposure to, for example, low temperatures, nutrient deficiency, and PTE in the soil, plants naturally and uncontrollably overproduce various types of reactive oxygen species (ROS) that can have a deleterious impact on plant metabolism. An imbalance between ROS production and their detoxification by enzymatic and non-enzymatic reactions represents metabolic states referred to as oxidative stress. As a result of higher net ROS formation, photo-oxidative damage to DNA, proteins, and lipids occurs and ultimately leads to cell death. Moreover, for example, low temperatures, constant darkness, and hypoxia can generate pathological levels of reactive nitrogen species (RNS), inducing nitrosative stress, and eventually leading to oxidative damage^16^. At low concentrations ROS and RNS act as signal molecules for the regulation of plant growth and development, and generate defense against biotic and abiotic stress^17^. During freezing and thawing, damage (rupture) to the plasma membrane can also occur due to the water expansion contained in the cells as it turns to ice, and the most damaging part of freezing injury is the severe cellular dehydration occurring upon ice formation and the destruction (tearing) of plant tissues^18^. Under temperate or continental climates, where rainfall is spread over the year, low temperatures are normally the main limiting factor for the growth of forage plants including some grass cultivars, and winter dormancy and/or cold tolerance are the main adaptative responses^19^. *Lolium perenne L*. has already been well-documented for its cold tolerance, interpreted as the ability to survive and function under cold conditions in temperatures such as non-freezing temperatures^20,21^. Based on the crop yield results observed by us, it can be deduced that *Lolium perenne L.*, placed in the freezing–thawing chamber in the HNT series, began winter dormancy naturally though faster after some time, saving energy and nutrients in its roots and other underground structures, remaining photosynthetically inactive, reducing water content and depressing their metabolic rate to survive unfavorable conditions than in the control series^22^. While *Lolium perenne L*. not exposed to low-temperature stress in a greenhouse was characterized by a higher yield as compared to the control series, which can be explained by the fact that the presence of HNTs in the soil indirectly helped in reducing the oxidative stress under metal stress by partly removing PTEs from contaminated soil, and better access to soil-soluble nutrients contributed to causing more favorable growth conditions^23^.

**Figure 2:**
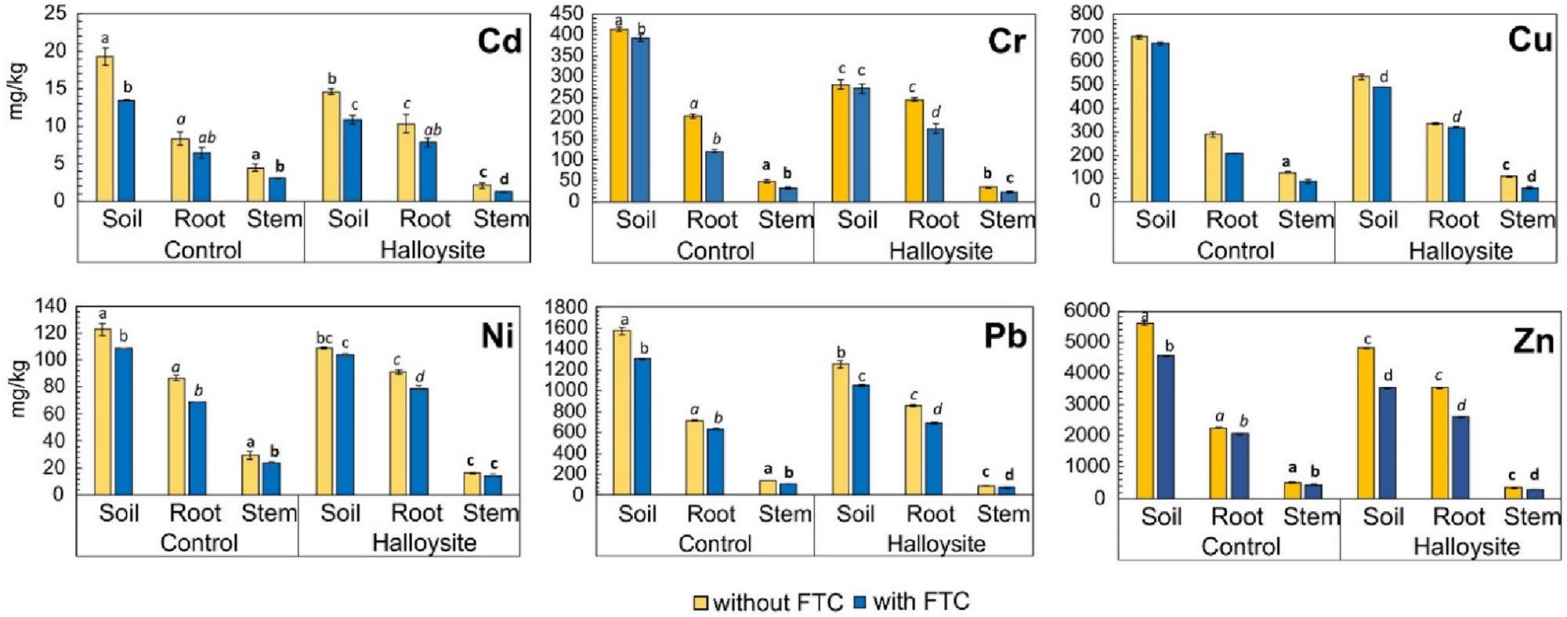
Changes in total contents of PTEs in not enriched (control) and halloysite-enriched soils, roots, and stems, without and with FTC. Different letters (normal letter for soil, italic letter for root, and bold letter for stem) indicate significant differences (*p* < 0.05) in PTE accumulation between soil, root, or stem. Data are shown as mean ± SD, n = 3.

The effective technique of aided phytostabilization comes down to taking up and accumulating PTEs in the roots of plants while, at the same time, maintaining their low contents in the above-ground parts, it is also called rhizostabilization^24^. Nevertheless, the bioavailability and toxicity of dissolved metal ions including harmful are not only related to their concentrations in soil water or groundwater but also, and primarily so, to their speciation^25^. Furthermore, speciation alone is insufficient for predicting accumulation and toxicity in plants because the presence of competing cations and soil organic matter (SOM provides specific acids, bases, and buffers) can affect the processes. Typically, 50% or less and 80% or more of dissolved organic matter (DOM) is considered to be composed of water-soluble acids, respectively, such as fulvic (at full pH range) and humic (at higher pH > 2), and these organic interactions with DOM accounts for mobility/immobility and bioavailability of nutrients and metals within the soil, making them soluble^26^. Due to its varied molecular composition, DOM can be highly reactive or quite recalcitrant but water content in the soil mainly determines what is in situ DOM^27^. Most studies confirm that metal ions are strongly covalently bound to DOM or through ion–dipole interactions, so the analogy may also apply to limit the uptake of PTE-ions by plants. Both, in the greenhouse as well as in the freezing–thawing chamber, DOM in the HNT-enriched series decreased max. by approx. 15% in relation to the appropriate control series, indicating specific HNT-DOM interactions. The natural HNTs can act in two-way in the soil because it is an excellent machine for capturing both types of charges, anions that are pushed into the tube’s lumen and cations that are adsorbed on the tube’s outer surface^28^. The outer and inner surface chemistry of HNTs is different, and the superposition of the tube’s negative (silica-sheet) outermost surface charges with positive (alumina-sheet) innermost surface charges is qualitatively interpreted as a net electric zeta-potential of HNTs, which can typically range up to − 30 mV and the charge separation for both surfaces (as the negative/positive charges) operates in the pH range of the surrounding medium between 2 and 8^29,30^. The net negative charge of HNTs increases with increasing pH in water solution, which is mainly attributed to the deprotonation of HNT-hydroxyls at high pH value. The lower the value of zeta-potential, the better the colloidal stability of HNTs in water, which ensures greater efficiency of ion species sorption, assuming also that HNTs (if present in soils, sediments, and deposits) mainly have particle sizes below 2 μm^31^. Most often, non-specific physical interactions (adsorption/desorption) occur with PTE-ions and/or organic molecules, e.g. from SOM, despite halloysite hydrophilic character^32^. By adsorbing to the surface of HNT particles, DOM in some amount became most likely immobilized and/or removed from the dissolved phase of the soil in our case. DOM removal can also result from microbial activity. In our study, in the soil microflora *Actinobacteria*, *Sphingomonas*, and *Planctomycetia*, were abundant in both phytostabilized soils (enriched with HNTs and not enriched). All these taxa metabolize organics present in soil and may have contributed to the reduction of DOM content. Generally, the adsorption performance of raw HNTs is mainly related to ion-exchange capacity, specific surface area (SSA), and porosity. The HNTs are endowed with cation-exchange capability mainly based on naturally deposited guests (in moderate to minor amounts) like alkali and alkaline earth cations, existing in the rolled interlayers or on the surfaces to balance the tube's net negative charge^33^. Besides that, the occurrence of various associated minerals with HNTs can significantly affect the obtained behavior of CEC. In our case, it stems from the abundance of Fe-rich and (Ti, Mn, P)-poor minerals. The specific forms of Fe, Ti, and P occurring with natural HNTs correspond to the geological environment of ’Dunino’ deposit. Related to this previous fact, the superposition of soil CEC and HNT CEC determines the soil’s true capacity to retain and exchange essential plant nutrients and promote plant growth, assuming that CEC_soil_ > CEC_HNT_^34–37^. Both, in greenhouse and chamber series, the content of analyzed metals in the roots was significantly higher than in the above-ground parts of *Lolium perene* L., with a clear tendency for greater accumulation of metals in the roots in the presence of HNTs in the soil (Fig. 2). The greatest accumulation of metals in the roots (as total contents) in the HNT series from the greenhouse was noted for Zn (56%), Cd (24%), Cr (20%), and Pb (19%), whereas from the freezing–thawing chamber—for Cu (53%), Cr (44%), Zn (27%) and Cd (22%) in relation to the appropriate control series. This can be explained by the fact that within the particular pH condition in the soil system, the solubility and bioaccessibility of PTE-ions are locally strongly influenced by the activity of plant roots, and can fluctuate particularly by the presence of a specific microbial community. Moreover, the impact of low temperatures can also naturally reduce the efficiency of plant roots and slow down the rate of uptake of soluble PTE-ions from the soil as well as lead to the dormancy of plant species^38^.

Regarding the effect of FTC exposure on the content of individual PTEs in roots, a less intense relative decrease in Cu by 5%, Ni by 13.7%, Cr by 28% contents in roots (in relation to the series enriched with HNTs and not exposed to FTC) is observed in the presence of HNTs than in its absence. And at the same time, a more intense relative decrease in Cd by 57%, Zn by 26%, Pb by 24% contents in roots (in relation to the series enriched with HNTs and not exposed to FTC) is observed in the presence of HNTs than in its absence. This indicates that effective removal only occurs for specific PTEs (Cu, Ni, Cr) by roots in the presence of HNTs in the soil upon freezing and thawing.

### HNT and FTC effects on soil pH and PTE content

**Figure 3:**
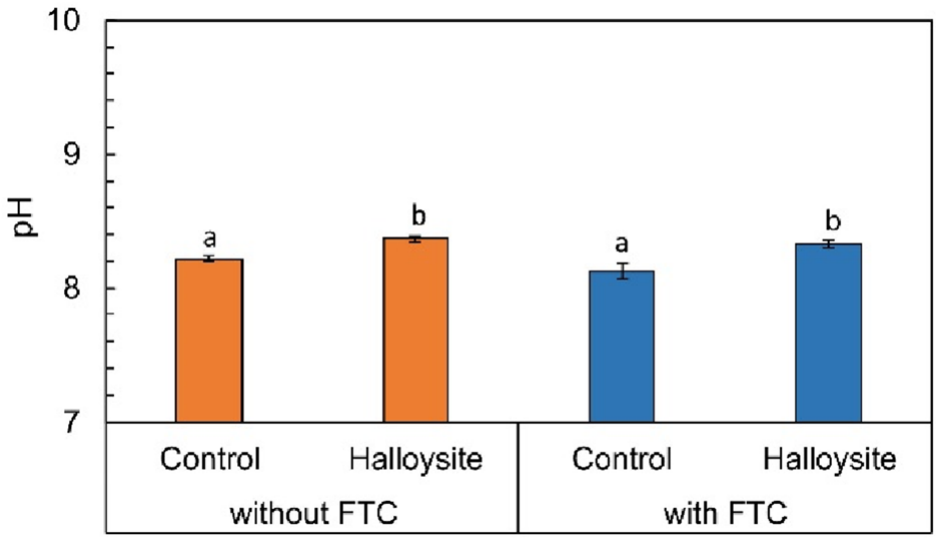
Changes in soil pH, without and with FTC in not enriched (control) and halloysite-enriched soils. Data are shown as mean ± SD, n = 3. Different letters indicate significant differences (*p* < 0.05) in pH of soil samples from different treatments.

The acidity of soil can lead to the release of easily soluble ionic forms of PTEs, which can have a harmful effect on plants and, in the case of the phytostabilization technique adversely affect the development of the vegetation cover. That is why the pH of the soil plays a key role. A significant increase in the pH value of soil can be obtained by applying alkalifying additives^2^. The studies by Neina^39^ reveal that the presence of specific plant cultivars as well as the activeness of microorganisms in the rhizosphere can lead to an increase in the pH of the soil by approx. 0.5 units, which also has a beneficial effect on the process of phytostabilization. In the carried out experiment, the addition of HNTs to the soil (Fig. 3) led to an insignificant increase in the value of soil pH by 0.16 (in the series not exposed to FTC) and by 0.21 units (in the FTC series) in relation to the respective control series. Notably, HNTs improved soil pH after FTC exposure relatively more than before FTC exposure. Regarding the effect of FTC exposure on soil pH, a more intense relative decrease in soil pH was observed in the control series by 1.1% (in relation to the soil not enriched with HNTs and not exposed to FTC) than in the HNT series by 0.5% (in relation to the soil enriched with HNTs and not exposed to FTC), indicating that the additive itself keeps pH in the soil almost constant even during freezing and thawing.

The application of natural HNTs into the soil can directly improve the processes of PTE-immobilization in the soil either by the additive itself (i.e., adsorption/desorption of metal ions on HNT surface) or by plant roots (i.e., absorption of metal ions). In the carried out experiment of aided phytostabilization the content of analyzed PTEs in the soil significantly depended on the addition of HNTs as well as FTC (Fig. 2). In the greenhouse series, the presence of HNTs most significantly lowered the contents of Cr (32%), Cd (24%) and Cu (24%) in the soil, whereas in the freezing–thawing chamber—of Cr (31%), Cu (28%) and Zn (23%) in relation to the appropriate control series.

### PTE distribution pattern and their stability in soil

**Figure 4:**
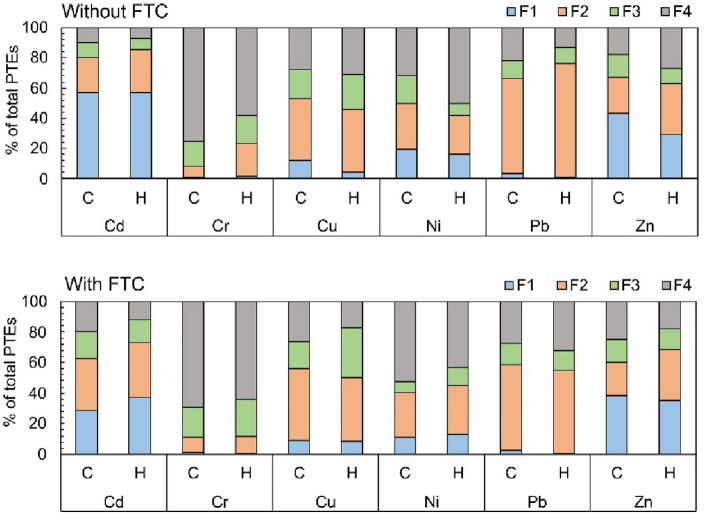
Changes in PTE distribution in soils without and with FTC (C denotes control soil, H denotes halloysite-enriched soil). Data are shown as mean ± SD, n = 3.

A comparison of PTEs distribution in not enriched soil (C) as control and HNT-enriched soil (H), without and with FTC, is shown in Fig. 4. The individual PTEs differed in their distribution. In the soils without FTC, Cd was highly mobile (~ 57% of the F1 fraction) in both the control and the soil enriched with HNTs. The presence of HNTs facilitated the redistribution of Cd to the F2 fraction. Cr was stable in the soil due to a high share of F4 fraction. The presence of HNTs increased the proportion of the F2 fraction and decreased the proportion of the F4 fraction of this metal ion. Compared to the control soil, HNTs led to a decrease in the proportion of Cu in the F1 fraction and a slight increase (by 3–4%) in the F3 and F4 fractions. The HNTs increased the stability of Ni by increasing the proportion of Ni in the F4 fraction in the soil. In the enriched soils, Pb had a higher proportion in the F2 fraction compared to the control soils. The obtained results confirmed a positive role of HNTs in Zn-immobilization by decreasing its mobility and increasing the PTE proportion in the F2 and F4 fractions. The high mobility of some PTEs was related to metal precipitation with carbonates. In our study, the soil samples had alkaline pH. The presence of carbonate minerals in soil results in the formation of double salt complexes with some PTEs such as Zn and Cd. For other metals, the greater effect on metal distribution in soil had oxides and clay minerals. At higher pH, surface hydroxyl groups are responsible for metal complexation on oxides, oxyhydroxides, hydroxides and aluminosilicates. The pH 6–8 seems to be optimum for metal adsorption onto clay minerals^40,41^.

**Figure 5:**
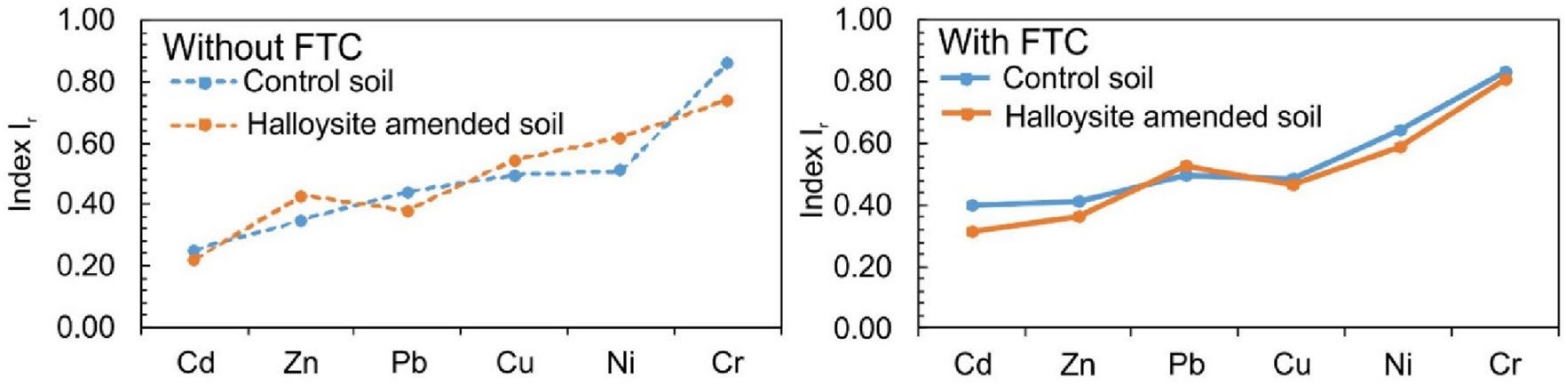
The effect of FTC and halloysite on the stability of PTE in soils based on the Ir index. Data are shown as mean ± SD, n = 3.

In the soils exposed to FTC, some changes in PTE distribution were observed. FTC further reduced mobility, but only for some PTEs such as Cd, Ni, and Pb. In HNT-enriched soil, the proportion of PTEs in the F2 fraction was higher than in enriched soil without FTC only for Cd and Ni. For other PTEs, the proportion of this fraction was decreasing (Cr, Pb) or unchanged (Cu, Zn). Some similar trends were observed in the control soils. The FTC resulted in an increase in the PTE content (by 3–9%) in the F3 fraction. Soil enriched with HNTs and exposed to FTC was most beneficial for increasing the fraction of F3 for Cr, Cu, and Ni. In HNT-enriched soil, FTC also further increased the proportion of Cd, Cr, and Pb in the F4 fraction, while decreasing this proportion for other PTEs. After FTC, the proportion of most PTEs (except Pb) in the F4 fraction was higher in the control soil than in the soil enriched with HNTs. This suggests that under FTC conditions, the role of halloysite in redistributing PTE to the most stable fraction may be limited. This was confirmed by Ir index values (Fig. 5). The Ir is the reduced partition index calculated for an individual metal based on its percentage content in specific fractions from the sequential extraction procedure. The Ir allows quantifying the metal binding intensity in the soil, which is related to the metal immobilization in the soil^42^. In soil without FTC, halloysite had a better effect on increasing the stability of Zn, Cu, and Ni than the control soil. However, in soil with FTC, higher Ir values for PTEs (except Pb) were reported for the control soil than for the enriched soil.

In this study, for most PTEs in HNT-enriched soil, the increase of PTE in the F2 fraction was observed. PTEs occurring in the reducible fraction are assumed to be mainly associated with Fe and Mn oxides and released upon the establishment of reducing conditions^43^. Depending on the origin, halloysite may contain various contents of iron compounds including iron oxides^44^, and the latter can decrease PTEs mobility by sorption or co-precipitation or by forming secondary minerals of PTEs^45^. Thus, during phytostabilization, some amounts of active PTEs (e.g. from F1 fraction or released by root activity in the rhizosphere) could be easily absorbed on the Fe/Mn oxides resulting in increasing the reducible fraction of PTEs. Since halloysite is an aluminosilicate clay mineral it can increase PTE content in residual fraction^46^, that was confirmed for Cu, Ni, and Zn. Our previous investigation on using halloysite as an amendment for soil from a former military area contaminated with multi-PTE ‘solution’ has revealed that this amendment increased to the highest extent (by 19–20%) the share of Cu and Ni in the F4 fraction and to a lesser extent for Pb and Zn^15^.

In both phytostabilized soils (control and HNT-enriched) the lowest stability was shown for Cd (Ir = 0.22–0.40) and the highest for Cr (Ir = 0.74–0.86), that was caused by the share of the most mobile fraction F1 and the share of the most stable fraction F4. The FTC caused an increase in the stability of most PTEs (Cd, Zn, Pb, Ni) in control soil and only Cd, Pb, and Cr in HNT-enriched soil compared to soil without FTC. After FTC the stability of Cd, Zn, Cu, Ni, and Cr was higher in control soil than in HNT-enriched soil. This may indicate that more changes in soil structure due to FTC in control soil than in enriched soil. It is known that repeated FTC can modify physical and chemical soil properties such as soil texture, surface area, cation exchange capacity, etc. that can facilitate PTE redistribution to increase their stability^47,48^. The literature data also indicate that soil amendments also are modified under FTC. Wang et al.^49^ demonstrated that after 30 FTC, the –OH and –COOH groups and surface area of biochar increased resulting in greater adsorption of Cu and Zn compared to pristine biochar. Since halloysite is one of the aluminosilicates with a layered structure, the FTC can also cause changes in its structure that enhance its sorptive properties and cause a redistribution of some PTEs^2^.

### HNT and FTC effects on soil microbiome

Using 16S rRNA gene amplicon sequencing, which generated approx. 160 k high-quality reads, the composition of the microbiome of the soil samples was examined (Table 1). The average yield of the high-quality DNA extraction was 264 ng/µl. The rarefaction curves indicated sufficient sequencing depth (data not shown). In the control sample after FTC, diversity decreased by approximately 8%. For the same soil enriched with HNTs after application of FTC, the Shannon index was the highest, reaching 5.92 (Table 1). This shows that HNTs have some relieving effects on microbial communities exposed to low temperatures and help maintain high microbial biodiversity. In diverse communities, there is a bigger chance that some species will adapt to environmental stress such as PTE presence. In a community with higher biodiversity, cooperation between microorganisms can maintain their high metabolic activity and ensure efficient pollutant removal. Halloysite has a unique nanotube structure, high porosity, and specific surface area, which can create an additional niche for microorganisms and protect them from the negative effects of environmental conditions.

**Table 1:** Alpha diversity indices for the analyzed soil samples.

Sample		Reads	Observed OTUs	Shannon	Chao1
Without FTC	Contr_bFTC	39,357	1138	5.73	1169
	Hal_bFTC	49,188	782	5.01	800
With FTC	Contr_aFTC	40,066	1028	5.29	1082
	Hal_aFTC	29,048	1101	5.92	1141

**Figure 6:**
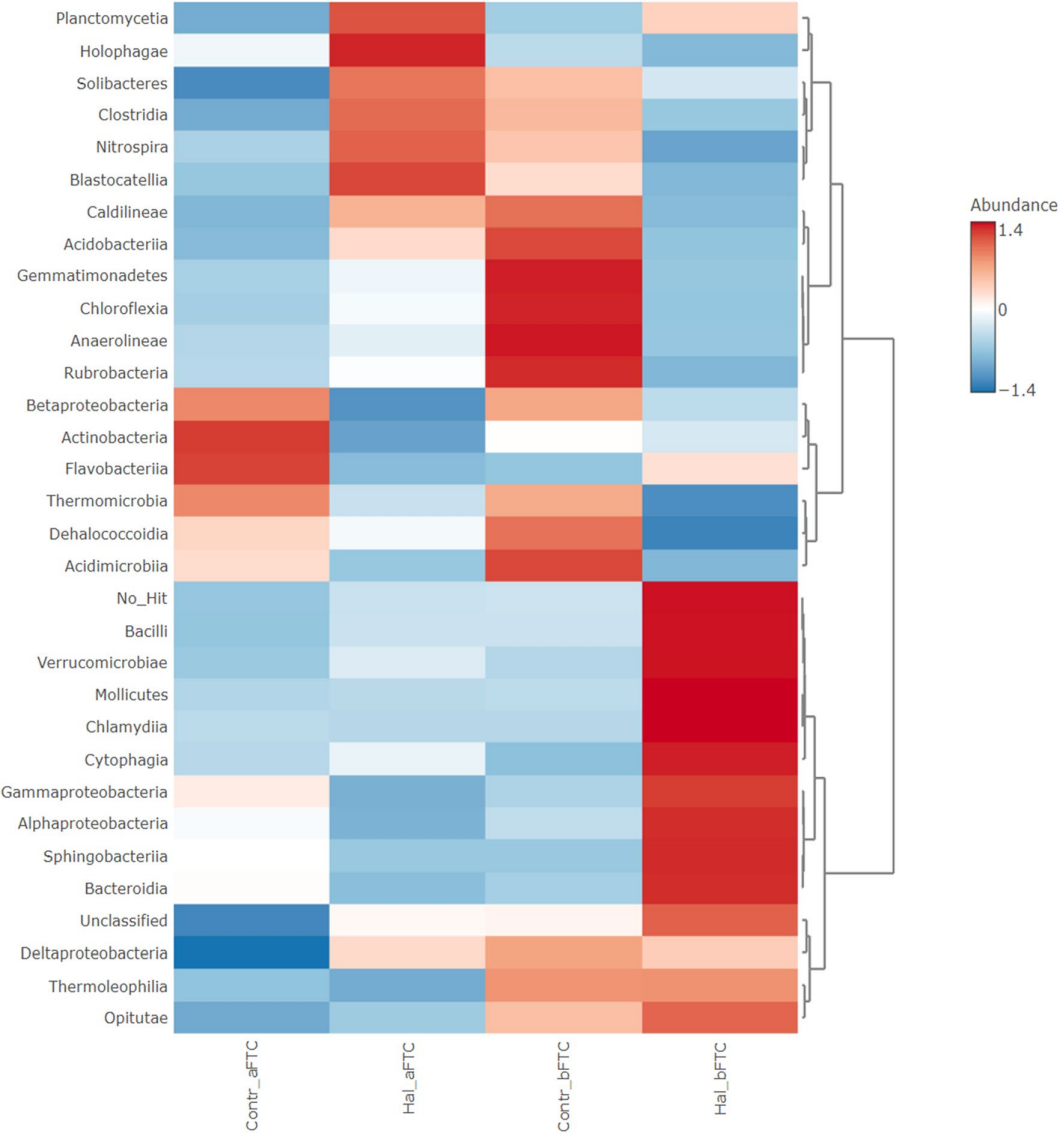
Heatmap presenting differences in the relative abundance of particular sequences among all obtained sequences between the not enriched (control) and the halloysite-enriched soil samples, respectively (class level Euclidean distance measure, Ward algorithm used for clustering).

Temperature changes are one of the most important environmental factors that affect the composition of microbial communities in soil and thus PTE biodegradation. At the class level, FTC decreased the abundances of *Opitutae* and *Thermoleophilia* in both control and HNT-enriched soil samples (Fig. 6). The microorganisms belonging to *Thermoleophilia* are actinobacterial species with very different morphologies and a variety of adaptations for surviving and thriving in hostile environments such as PTE-contaminated soil^50^. However, they are thermophilic or thermotolerant species. Members of *Opitutae* also prefer higher temperatures^51^. This could explain the lower abundance of bacteria belonging to these two classes in the soil after FTC. No abundant microbial classes were identified whose abundance increased in both the control and HNT-enriched samples after FTC treatment, suggesting that there is no clear trend in the microbial structure after FTC treatment. In the control sample, the abundance of *Actinobacteria* and *Flavobacteriia* increased after FTC. As an important component of the soil microbiome, *Actinobacteria* are involved in carbon cycling and soil organic matter formation^50,52^. By cooperating or cross-feeding with other soil bacterial species, *Actinobacteria* are able to continue their metabolic activities even at low temperatures^53^. According to a study by Navas et al.^54^, *Actinobacteria* are widely distributed in soils with low metal(loid) contamination.

This study increases knowledge on how the exposition of soil to amendment affects the soil microbiome. A lack of additives in the soil stimulated the growth of microorganisms belonging to *Betaproteobacteria*, *Thermomicrobia*, *Dehalococcoidia*, and *Acidimicrobia*, while the amendment of PTE-contaminated soils with HNTs increased the abundance of *Planctomycetia*. Studies relating the potential ecological risk index (RI), the most commonly used method for assessing PTE contamination in soils, to microbial structure showed that *Planctomycetes* were enriched with increasing RI^55^. Rare taxa, including *Planctomycetes*, play important roles in metal(loid) metabolism and functional diversity^56^ and can respond rapidly to maintain community stability when faced with PTE stress^57^. *Planctomycetes* can secrete extracellular polymeric substances that may have helped colonize halloysite nanotubes in the enriched soil. Extracellular polymers have also been reported to aid in the detoxification of PTEs^58^. In the presence of HNTs, an abundance of bacteria of the classes *Holophagae*, *Solibacteres*, *Clostridia*, *Nitrospira*, and *Blastocatellia* increased after FTC compared to the control sample. In the enriched sample not treated with FTC, *Bacilli*, *Verrucomicrobiae*, *Mollicutes*, *Chlamydia*, *Cytphagia*, *Gammaproteobacteria*, *Alphaproteobacteria*, *Sphingobacteria*, and *Bacteroidia* were most abundant. FTC decreased the occurrence of bacteria from these classes in the soil.

The analysis of sequencing results indicated that in all investigated soils some core microbial genera typical for PTE-contaminated soils were identified such as *Sphingomonas* sp. (over 3%), *Acidobacterium* sp. (ca. 1–3%), and *Mycobacterium* sp. (ca. 2–3%) (Table 2). These taxa were found in soils contaminated with Co^2+^, Ni^2+^, and Cd^2+^ and were involved in their transformation^59^. The presence of these bacterial genera in the soil is beneficial because their metabolic activity can gradually improve soil properties and promote the development of bacteria with more demanding environmental requirements^52,60^. According to previous studies, *Mycobacterium* sp. and *Sphingomonas* sp. thrive in PTE-contaminated soils^54,59^, likely due to their PTE resistance genes^61,62^.

**Table 2:** 26 genera with the highest percentage abundances in the analyzed soil samples.

	Contr_bFTC (%)	Contr_aFTC (%)	Hal_bFTC (%)	Hal_aFTC (%)
*Acidobacterium*	2.33	1.09	1.14	3.48
*Alkanindiges*	0.00	5.68	0.11	0.00
*Arthrobacter*	3.11	1.26	0.63	2.68
*Blastococcus*	1.10	0.52	0.33	0.39
*Bradyrhizobium*	0.85	0.89	0.73	0.64
*Brevundimonas*	0.34	0.43	1.75	0.11
*Candidatus_Microthrix*	0.61	1.70	0.29	0.20
*Herbaspirillum*	1.75	1.89	0.63	0.79
*Hyphomicrobium*	0.61	0.31	0.51	1.13
*Iamia*	1.49	0.89	0.23	0.67
*Massilia*	2.25	1.21	0.45	0.46
*Mycobacterium*	3.66	2.78	2.35	1.94
*Nocardioides*	0.97	2.46	1.33	0.80
*Ohtaekwangia*	0.61	0.54	1.14	1.58
*Pelobacter*	1.33	0.39	0.66	1.28
*Phenylobacterium*	0.63	0.68	1.48	0.29
*Prochlorococcus*	0.74	0.45	1.67	1.02
*Pseudoxanthomonas*	0.19	0.50	2.14	0.51
*Ramlibacter*	0.91	1.01	0.67	0.56
*Rhodococcus*	1.01	5.60	0.26	0.41
*Rhodoplanes*	0.97	0.74	0.32	0.72
*Sphaerobacter*	1.00	1.10	0.18	0.89
*Sphingomonas*	3.58	3.38	3.16	3.46
*Terrimonas*	0.59	0.60	0.84	0.84
*Thermomonas*	0.20	2.49	0.15	0.09
*Williamsia*	0.87	4.45	1.11	0.08

In not enriched soils exposed to FTC, a high percentage of bacteria belonging to the genera *Rhodococcus*, *Williamsia*, *Thermomonas*, *Alkanindiges*, and *Nocardioides* were observed (Table 2). These genera are characterized by tolerance to PTEs in the environment^59,63^. *Rhodococcus* sp. with one of the most robust resistances to PTEs^64^ is among the most promising genera for bioremediation studies due to the presence of genes and pathways for metal transformation in their genomes.

In conclusion, this work contributed to the improvement of knowledge in the process of thermal treatment involving freezing and thawing of soils cultivated with plants and in the presence of microbiome, which is an important step in PTE immobilization in assisted phytostabilization techniques. HNT addition into the soil increased the above-ground grass biomass, and in its presence a more intense relative increase was observed after freeze–thaw cycles. PTE accumulation in grass roots was greater than in the above-ground parts, with HNT presence in soil, both in a greenhouse and chamber conditions. HNT addition significantly increased Zn accumulation in roots; however, an almost two-fold relative decrease was observed after freeze–thaw cycles, and an almost two-fold relative increase was observed for Cu and Cr. On the other hand, FTC effect indicates that only specific PTEs, such as Cu, Ni, Cr were effectively removed through the roots in HNT presence even during freezing and thawing. HNT presence slightly increased soil alkalinity, and helped to keep it almost constant even during freezing and thawing. HNTs have a positive effect on PTE stability, improve the immobilization of Zn, Cu, and Ni in stable fractions (reducible and residual), and increase the values of the reduced partition index (Ir). However, this effect decreases under FTC exposure, which illustrates the complex interplay between HNTs, FTC, and soil properties in shaping PTE distribution. HNT presence helps to maintain high microbial biodiversity in soils exposed to low temperatures. The amendment of PTE-contaminated soils with HNTs stimulated the growth of *Planctomycetia* sp.

## Methods

### Site, source soil preparation, and description

The site comprised an industrially PTE-contaminated area selected based on the analysis of the level of PTE contents, significantly exceeding the maximum levels indicated by the Polish law^65^. The soil, approx. 80 kg, was removed from the surface layer of the terrain (0–0.25 m) located in central Poland from a few 1 × 1 square meter quadrants. It was next dried under laboratory conditions at room temperature (72 h) and sieved through a 2 mm sieve. The most important soil properties were indicated: pH (8.92 ± 0.18), total soil organic matter content % (SOM) (1.17 ± 0.11), cation exchange capacity Cmol/kg soil (CEC) (54.2 ± 0.7), total PTE contents mg/kg soil (Cd 33 ± 1.15, Ni 251 ± 7.18, Cr 687 ± 11.20, Cu 1155 ± 34.34, Pb 2486 ± 35.31, Zn 8481 ± 95.34). Based on the individual percentage shares of the granulation of the soil and the USDA soil texture classes, it was classified as loamy sand (72.0% sand, 26.7% silt, 1.3% clay)^66^.

### Halloysite description

The Polish halloysite from ‘Dunino’ opencast mine near Legnica consists of predefined nano-sized structures in the form of plates (HNP) with a flat and partially rolled surface and tubes (HNT) formed of rolled plates. All three geometric forms occur simultaneously in nature and contribute to overall properties, although halloysite is mostly considered to be nanotubes. Notably, the high purity of the deposit and the morphology of halloysite grain are related to its local origin, and in particular to the crystallization conditions existing in the geological environment. It is usually composed of silica, alumina, and water, frequently with moderate to minor amounts of iron, alkali and alkaline earth metals^67^. The most important properties were indicated for raw halloysite: specific surface area (SSA_BET_) (52.5 m^2^/g); at pH (max. 12.1) the reported CEC is (max. 17.2 Cmol/kg)^33^. Morphology was studied with SEM, and the elongated-tubular structure was confirmed^68^. Before FTC exposure, the elemental composition reveals main constituents in the form of elements as an average %, w/w (Si-23.38, Al-20.38, and Fe-9.50); while elements deposited in small amounts are (Ti-1.15, Na-1.04, Ca-0.90, Mg-0.71, K-0.28, Mn-0.26 and P-0.41); negligible content was found for Cu–0.04; and none (Cd, Ni, Cr, Pb, Zn). The raw material was initially milled and sieved using a 0.05 mm mesh to obtain a fine powder.

### Experiment details

Halloysite-aided phytostabilization was carried out as a pot experiment in two separate temperature variants: (1) in a greenhouse (before i.e. without freeze–thaw cycling FTC) and (2) in a freezing–thawing chamber (after i.e. with FTC). For this aim, 6 pots each of which was filled with 5 kg of contaminated soil (control), and 6 pots which were filled with soil enriched with halloysite at 3.0% (w/w). As a test plant, *Lolium perenne* L. was applied, which was sown in the amount of 0.005 kg per pot. The greenhouse experiment lasted 52 days, while controlling the temperature of the environment (day 26 ± 2 °C, and night 16 ± 2 °C) and the moisture content of the soil maintained at a level of approximately 60% of the maximum water holding capacity. Upon completing the greenhouse experiment, the plants from half the pots were cut (separating the roots from the above-ground parts) and soil samples were taken for further indications. The remaining pots with plants were placed in a chamber (Toropol K-010) for a period of 64 days and subjected to 16 FTC cycles. In our study, selected parameters and values, such as time, temperature, and the number of freeze–thaw cycles were selected based on Hou's methodology^69^. The original Hou's methodology involved the determination of metal transformations in Cd/Pb-contaminated soil using various soil additives under FTC exposure, while in our experiment, *Lolium perenne* L. was cultivated simultaneously in soil enriched with HNT additive. The extension of the soil and additive system to include plant cover and microbial genera enabled us to use it as an integrated system in phytostabilization. In an individual cycle, the temperature in the chamber changed periodically between the freezing and thawing phase. Freezing lasted 48 h at − 20 ± 0.5 °C and a relative moisture content of 90 ± 10%, thawing 48 h at 20 ± 0.5 °C and a relative moisture content of 50 ± 10%, at an ambient pressure of approx. 1000 ± 10 hPa. The experiment in the chamber was completed after 116 days (52 days in the greenhouse and 64 days in the chamber). Further plants and soils were taken for indications. The study of the transition at a specific time, i.e. from summer to winter, involved adapting the experimental setting to weather conditions, time and age of the plants. Hence, aided phytostabilization was carried out in two temperature variants, first in a greenhouse and then in a freezing–thawing chamber.

### Physico-chemical analysis of plant biomass, additive, and soil

The particle size distribution of the source soil was measured (Mastersize 2000, Malvern Panalytical). The measurement of soil pH and halloysite were carried out in deionized water at a ratio of 1:2.5 w/v (pH-meter HI 221, Hanna Instrument). Plant materials were digested in a mixture of 65% HNO_3_ and 30% H_2_O_2_ whereas soil materials were sieved through a 1 mm sieve and digested in a mixture of 36% HCl, 65% HNO_3_, and 30% H_2_O_2_ (microwave oven, MARSXpress). FAAS was used to measure the levels of Cd, Ni, Cr, Cu, Pb, and Zn (280FS AA, Varian) in mineralization analytes and in four target fractions obtained from the modified BCR (Community Bureau of Reference) sequential extraction of soils, such as exchangeable, acid- and water-soluble F1, reducible F2, oxidizable F3, and residual F4^70^. CEC values of soil materials were calculated in each case as the sum of hydrolytic acidity (HAC) (in 1 M Ca(CH_3_COO)_2_) and total exchangeable bases (TEB) (in 0.1 M HCl); a variation of the Kappen-Roedder method^71^. The halloysite CEC value using the cobalt hexamine trichloride [Co(NH_3_)_6_]Cl_3_ method was reported by Ciesielski et al.^33^, Gray et al.^36^. Total SOM content was determined by soil burning at 550 °C (muffle furnace ESM 9920, Carbolite). Soil DOM was extracted by shaking soil in deionized water at 200 rpm and 20  C for 2 h. The suspension was filtered through 0.45 μm polyether sulfone membrane (Millipore) and stored at 4 C before further analyses. Nitrogen adsorption at 77 K was used to determine halloysite SSA_BET_ (ASAP 2020, Micromeritics). SEM–EDX was used to study halloysite morphology and elemental composition of the surface (LEO 1430 VP, Jeol).

### Microbial community analysis

A FastDNA SPIN Kit for Soil (MP Biomedicals) was used to extract the genomic DNA from 500 g of soil from each experimental repetition. A NanoDrop spectrometer was used to evaluate the purity and concentration of the soil DNA that had been isolated from each experimental repetition (Thermo Scientific). DNA from the same experimental run was mixed and used for amplification with a universal primer set that targets both bacterial and archaeal 16S rRNA genes, 515F/806R (5'-GTGCCAGCMGCCGGTAA-3′/5′-GGACTACHVGGGTWTCTAAT-3′^72^. The amplicons were sequenced using the Illumina MiSeq platform in the same run (Research and Testing Laboratory USA). The readings' bioinformatical processing is detailed in Radziemska et al.^73^. The sequencing data have been deposited in Sequence Read Archive (SRA, NCBI) as BioProject PRJNA777436. MicrobiomeAnalyst^74,75^ was employed for statistical and meta-analysis of soil microbiome data (*p* < 0.05). Low-abundance bacteria may play a significant role in complex microbial consortia. To maintain the highest precision level for each sample, the number of reads was not standardized before the calculation of diversity indices^76^.

### Statistical analysis

Statistical processing of data concerning selected soil properties, PTE distribution in soil, and metal accumulation in plant above-ground parts and roots was performed using Statistica 13.3 software. When significant differences in data were identified by ANOVA, further analysis by Tukey's HSD test was carried out.

## Data Availability

The datasets generated during and/or analysed during the current study are available from the corresponding author on reasonable request.
