# Author Correction: Effect of freeze–thaw manipulation on phytostabilization of industrially contaminated soil with halloysite nanotubes

**DOI:** 10.1038/s41598-024-54206-6

**Published:** 2024-02-13

**Authors:** Maja Radziemska, Mariusz Z. Gusiatin, Agnieszka Cydzik‑Kwiatkowska, Aurelia Blazejczyk, Grzegorz Majewski, Iwona Jaskulska, Martin Brtnicky

**Affiliations:** 1 Institute of Environmental Engineering, Warsaw University of Life Sciences, 02‑776 Warsaw, Poland; 2 Faculty of Geoengineering, University of Warmia and Mazury in Olsztyn, 10‑719 Olsztyn, Poland; 3 Institute of Civil Engineering, Warsaw University of Life Sciences, 02‑776 Warsaw, Poland; 4 Faculty of Agriculture and Biotechnology, Bydgoszcz University of Science and Technology, 85‑796 Bydgoszcz, Poland; 5 Department of Agrochemistry, Soil Science, Microbiology and Plant Nutrition, Mendel University in Brno, 613 00 Brno, Czech Republic

Correction to: *Scientific Reports* 10.1038/s41598-023-49698-7, published online 13 December 2023

The Acknowledgements section in the original version of this Article was incomplete.

“The research leading to these results received funding from Polish National Science Centre (MINIATURA 3) under Grant Agreement No. 2019/03/X/NZ9/01276.”

now reads:

“The research leading to these results received funding from Polish National Science Centre (MINIATURA 3) under Grant Agreement No. 2019/03/X/NZ9/01276. The publication was (co)financed by Science development fund of the Warsaw University of Life Sciences—SGGW.”

The original Article has been corrected.

